# Updates on intratumoral therapies in melanoma

**DOI:** 10.1002/cncr.70400

**Published:** 2026-06-25

**Authors:** Vincent T. Ma, Alyssa K. Steimle, Janmesh D. Patel, Caroline Burkey, Justin C. Moser, Asad Javed, Hibba tul Rehman, Mustafa Ege Seker, Alexander Birbrair, Orhan S. Ozkan, Mark R. Albertini, Yana G. Najjar, Rajan P. Kulkarni

**Affiliations:** ^1^ Department of Medicine University of Wisconsin‐Madison Madison Wisconsin USA; ^2^ Division of Hematology, Medical Oncology, and Palliative Care, Department of Medicine University of Wisconsin‐Madison Madison Wisconsin USA; ^3^ Department of Dermatology University of Wisconsin‐Madison Madison Wisconsin USA; ^4^ HonorHealth Research Institute Scottsdale Arizona USA; ^5^ Arizona State University School of Medicine and Advanced Medical Engineering Scottsdale Arizona USA; ^6^ University of Chicago Medicine Chicago Illinois USA; ^7^ Department of Medicine University of Maryland School of Medicine Baltimore Maryland USA; ^8^ Department of Radiology University of Wisconsin‐Madison Madison Wisconsin USA; ^9^ Medical Service William S. Middleton Memorial Veterans Hospital Madison Wisconsin USA; ^10^ University of Pittsburgh Medical Center Hillman Cancer Center Pittsburgh Pennsylvania USA; ^11^ Department of Dermatology Oregon Health and Sciences University Portland Oregon USA

**Keywords:** immunotherapy, intratumoral therapy, melanoma, oncolytic virus, talimogene laherparepvec, tumor microenvironment

## Abstract

Intratumoral therapies provide an opportunity for novel strategies in the management of advanced melanoma. These approaches deliver concentrated doses of therapeutic immune agents directly into individual tumor(s), achieving local tumor control and induction of systemic antitumor immune responses while minimizing the risk of immune‐related adverse events. Talimogene laherparepvec, a genetically modified herpes simplex virus type 1–encoding granulocyte‐macrophage colony–stimulating factor, was the first oncolytic viral therapy approved in a solid tumor indication. In pivotal trials of patients with melanoma, talimogene laherparepvec demonstrated durable responses. Building on this paradigm, next‐generation oncolytic agents incorporate additional genetic modifications, such as fusogenic proteins and immunostimulatory transgenes, to enhance local cytotoxicity and systemic immune priming. Clinical studies of intratumoral, injectable agents since 2010 have evaluated monotherapy as well as combination strategies with immune checkpoint inhibitors, revealing improved response rates and durable remissions, including in patients with immune checkpoint inhibitor‐resistant disease. This review summarizes the mechanistic rationale, preclinical evidence, and clinical development of intratumoral therapies in melanoma. Key trial outcomes, safety profiles, and practical considerations for administration are discussed. Emerging trends include image‐guided visceral injection, combinatorial regimens, and the integration of intratumoral therapy in the neoadjuvant setting. As these therapies evolve, intratumoral approaches may expand their role in melanoma management, bridging locoregional tumor control with systemic immune activation and complementing existing systemic treatments.

## Abstract 2

Intratumoral therapies deliver immune‐activating agents directly into tumor lesions, remodeling the tumor microenvironment to enhance antigen presentation and prime systemic antitumor immunity; these approaches include oncolytic viruses, pattern‐recognition receptor agonists, cytokines, messenger RNA, and small molecules. This review synthesizes mechanisms of action, clinical efficacy, and safety across leading platforms and proposes a roadmap for clinical trial end points, rational combinations with immune checkpoint inhibitors, neoadjuvant applications, and biomarker‐guided patient selection to support integration into melanoma management.

## Plain Language Summary

- The purpose of delivering immune‐active agents/medications directly into tumors (*intratumoral therapies*) is to change the tumor's environment, trigger an immune response, and create antitumor immunity.
- This review describes the methods, clinical effectiveness, and safety of intratumoral therapies; outlines methods of delivery and objectives of intratumoral therapies; and proposes a roadmap for combining these therapies with other immunotherapy drugs and patient‐selection criteria to inform integration into care for patients with melanoma.

## INTRODUCTION

Malignant melanoma is an aggressive skin cancer with rising incidence, responsible for approximately 100,000 new cases and greater than 8000 deaths in the United States in 2025.^1^ Although early stage melanoma is often curable with surgery, advanced disease remains associated with significant morbidity and mortality.^2^, ^3^ The advent of immune checkpoint inhibitors (ICIs), BRAF‐MEK inhibitors, and cellular therapies have markedly improved outcomes; however, treatment resistance and therapy‐related toxicity remain common.^4^ Intratumoral therapy has emerged as a promising approach by enabling the direct delivery of therapeutic agents into tumors, thereby achieving high local drug concentrations, minimizing systemic toxicity, and modulating the tumor microenvironment (TME) to promote local and systemic antitumor immune responses.^5^

Multiple intratumoral agents for treatment of melanoma act through immunologic signaling within the TME. Oncolytic viruses trigger tumor cell lysis and release of tumor‐associated antigens, promoting dendritic cell–mediated cytotoxic T‐cell priming, whereas cytokines and pattern‐recognition receptor agonists amplify similar immunologic pathways to generate local and systemic antitumor effects.^6^ The concept of intentionally introducing pathogens to induce an immune response that causes tumor reduction and eradication emerged in the 1800s, when physicians observed that patients with cancer developed tumor regression in the setting of systemic infection.^5^ In 2015, talimogene laherparepvec (T‐VEC) became the first US Food and Drug Administration‐approved oncolytic virus for the treatment of melanoma, representing the first regulatory approval for an oncolytic virus in a solid tumor indication.^7^, ^8^ Next‐generation platforms are now being developed with enhanced cytotoxicity and immune activation to broaden efficacy to systemic (visceral) and ICI‐resistant disease.^9^, ^10^, ^11^

This review provides an overview of intratumoral therapies for melanoma, emphasizing the mechanisms, clinical development, and practical considerations for investigational agents.

## TUMOR MICROENVIRONMENT MODULATION

The TME is often immunosuppressive, characterized by the presence of regulatory T cells, myeloid‐derived suppressor cells, and immunosuppressive cytokines.^12^, ^13^ Intratumoral therapies have the potential to modulate the TME by inducing immunogenic cell death, releasing tumor antigens, reducing/reprogramming T‐regulatory cells, and promoting the infiltration of effector immune cells. This reprogramming of the TME can enhance the effectiveness of systemic therapies and potentially lead to the regression of distant metastases.^12^

### Oncolytic viruses as immunotherapeutic agents

Oncolytic viruses selectively replicate in tumor cells, inducing lysis and release of tumor antigens that stimulate dendritic cell–mediated T‐cell responses. Advances in genetic engineering have improved tumor selectivity, immunogenicity, and modulation of the TME, including effects on neovascularization.^14^, ^15^ Clinical development has progressed from early safety studies to phase 2–3 trials, both as monotherapy and in combination with ICIs. Current approaches include synthetic viral mimetics, plasmid‐based cytokine delivery, and combination strategies to enhance local and systemic antitumor immunity.

#### Talimogene laherparepvec

T‐VEC is a genetically modified herpes simplex virus (HSV) type 1 that expresses granulocyte‐macrophage colony–stimulating factor (GM‐CSF). It preferentially infects and lyses tumor cells, releases GM‐CSF, recruits dendritic cells, and primes tumor‐specific T cells that can drive systemic immunity.^16^

The phase 3 OPTiM trial of intratumoral T‐VEC (ClinicalTrials.gov identifier NCT00769704) significantly improved durable and objective response rates (ORRs) over GM‐CSF in unresectable, stage III–IV melanoma with cutaneous or nodal disease, with durable response rate of 19.3% versus 2.0% and complete responses (CRs) in 16.9% of patients.^7^ The final analysis in 2019 demonstrated an improvement in progression‐free survival (PFS) and overall survival (OS), particularly among patients who had earlier, lower burden disease, with generally mild, flu‐like adverse events (AEs).^7^, ^8^ A meta‐analysis of eight studies (*n* = 642), including OPTiM, reported a median ORR of 52.5% and a durable response rate of 38%, with markedly higher response rates in patients who had stage IIIB–IV (M1a) disease (CR rate, 30%; ORR, 44%) versus those who had stage IIIB–IV (M1c) disease (CR rate, 4%; ORR, 9%). Grade 3–4 toxicities were comparable across studies.^17^

In a phase 2 trial of patients who had resectable, stage IIIB–IVM1a melanoma, neoadjuvant T‐VEC followed by surgery significantly improved 2‐year recurrence‐free survival (RFS; 29.5% vs 16.5%; hazard ratio [HR], 0.75) compared with surgery alone.^18^ OS benefits and pathologic CRs were also observed. A key limitation is that earlier exposure to systemic therapy may have given a biased advantage to the neoadjuvant T‐VEC arm, although the surgery arm received more subsequent anticancer therapies.

In a randomized phase 2 study of 198 individuals who had with anti–programmed cell death 1 (anti–PD‐1)–refractory, unresectable stage III–IV melanoma, treatment with T‐VEC and ipilimumab improved antitumor activity. The ORR was 35.7% in the T‐VEC plus ipilimumab arm compared with 16.0% in the ipilimumab‐alone arm. Reductions occurred in injected and visceral lesions, including 52% reductions in the combination therapy arm and 23% in the ipilimumab monotherapy arm.^19^, ^20^ Grade 3 or greater events occurred in 45% of the combination arm and 35% of the monotherapy arm.^19^, ^20^

Combining T‐VEC with anti–PD‐1 therapy has yielded mixed results.^21^ In the phase 2 Southwest Oncology Group S1607 trial of patients with melanoma who were refractory to prior PD‐1/programmed death ligand 1 (PD‐L1) therapy (ClinicalTrials.gov identifier NCT02965716), T‐VEC plus pembrolizumab demonstrated activity in patients those without visceral disease (ORR, 26%; two CRs, five partial responses [PRs]) but no confirmed responses in those with visceral metastases.^22^ In contrast, the phase 3 MASTERKEY‐265 trial (*n* = 692; ClinicalTrials.gov identifier NCT02263508) demonstrated no significant improvement in PFS or OS with T‐VEC plus pembrolizumab versus pembrolizumab alone (median PFS, 14.3 vs. 8.5 months; HR, 0.96; *p* = .74). Although the ORR (48.6% vs. 41.3%) and the durable response rate (42.2% vs. 34.1%) were numerically higher with combination therapy, these differences were not statistically significant, and the safety profiles were comparable.^19^, ^20^

Important limitations remain with T‐VEC. OS gains have been modest,^19^, ^20^ efficacy is lower in patients with later stage disease and higher burden of disease,^17^ and the label is restricted to unresectable cutaneous, subcutaneous, or nodal melanoma after initial surgery.^23^ Intratumoral delivery requires accessible, injectable lesions. Although selected deep visceral targets can be treated with image‐guidance, clinical experience is largely limited to cutaneous, subcutaneous, and nodal disease. T‐VEC is contraindicated in pregnancy and in immunocompromised individuals because of herpetic risk.^24^ As of 2025, ongoing trials include: the phase 2 NIVEC study (neoadjuvant T‐VEC plus nivolumab; EU Drug‐Regulating Authorities Clinical Database [EudraCT] identifier 2019‐001911‐22),^18^, ^25^ the MASTERKEY‐115 phase 2 trial (T‐VEC plus pembrolizumab; ClinicalTrials.gov identifier NCT04068181) in anti‐PD‐1–refractory melanoma,^26^ and phase 1 and 2 studies across multiple other solid tumors.^26^, ^27^, ^28^, ^29^, ^30^, ^31^, ^32^, ^33^

#### Vusolimogene oderparepvec (RP1 and RP2)

Vusolimogene oderparepvec (RP1) is an oncolytic HSV type 1 virus engineered to express GM‐CSF and to enhance fusogenic activity through incorporation of GALV‐GP‐R− (gibbon ape leukemia virus envelope glycoprotein). RP1 permits a larger intratumoral injection volume compared with T‐VEC, enabling the treatment of more and larger tumor lesions. This modification allows RP1 to form syncytia, leading to enhanced tumor cell lysis and tumor antigen release.^9^

In the phase 2 IGNYTE trial (ClinicalTrials.gov identifier NCT03767348), intratumoral RP1 in combination with nivolumab demonstrated robust and durable efficacy in anti–PD‐1–refractory melanoma, with an ORR of 33% (15% CRs) by both modified Response Evaluation Criteria in Solid Tumors (RECIST) and RECIST version 1.1.^9^ Responses were durable (median, 22–28 months), with >85% ongoing beyond 1 year, and were observed across subgroups, including patients who had prior exposure to anticytotoxic T‐lymphocyte–associated protein 4 (anti–CTLA‐4) or primary PD‐1 resistance. The median OS was not reached (3‐year OS, 54.8%), and treatment was well tolerated (grade 3–4 events, 12%).^9^ Translational analyses showed increased CD8‐positive T‐cell infiltration, PD‐L1 upregulation, and inflammatory gene signatures.^34^

At the time of publication, factors influencing the initial US Food and Drug Administration approval of RP1 with nivolumab included challenges related to end point selection, regulatory expectations in the anti–PD‐1–refractory setting, and the need for robust, randomized, phase 3 data to demonstrate durable clinical benefit. A phase 3 randomized controlled study (IGNYTE‐3) of RP1 plus nivolumab versus physician’s choice (nivolumab plus relatlimab, anti–PD‐1 monotherapy rechallenge, or single‐agent chemotherapy) in patients who progressed on anti–PD‐1 plus anti–CTLA‐4 therapy is actively enrolling (ClinicalTrials.gov identifier NCT06264180).^35^ At the time of publication, RP1 remains investigational.

RP2, an HSV‐1 virus engineered to express GM‐CSF, a fusogenic glycoprotein (GALV‐GP‐R−), and an anti–CTLA‐4–like molecule, was evaluated in a phase 1/2 study (ClinicalTrials.gov identifier NCT04336241), as monotherapy and in combination with nivolumab in 17 patients with metastatic uveal melanoma.^36^ Most patients were heavily pretreated, including prior anti–PD‐1 and anti–CTLA‐4 therapy. RP2 alone and combined with nivolumab demonstrated promising activity, with an ORR of 29.4% (all were PRs) and a disease control rate of 58.8%, with responses lasting a median of 11.5 months. Treatment was well tolerated, with mainly grade 1–2 AEs; no grade 4–5 events occurred. At the time of this publication, the RP2 REVEAL trial (ClinicalTrials.gov identifier NCT06581406) is an active and enrolling, phase 2/3, adaptive study comparing RP2 plus nivolumab versus ipilimumab plus nivolumab as the control arm (a clinically relevant, standard comparator) in the treatment of ICI‐naive metastatic uveal melanoma.^37^

#### OrienX010

OrienX010 is a genetically modified HSV‐1 encoding human GM‐CSF that demonstrated a 19.2% ORR in a phase 1b monotherapy study of 26 patients with acral melanoma.^38^ Systemic immunogenicity was also observed, with responses in 54.6% of injected lesions and 48.8% of noninjected lesions.^39^ In a neoadjuvant phase 1b trial, 30 patients with stage III/IV acral melanoma received OrienX010 plus toripalimab preoperatively followed by adjuvant toripalimab alone for 1 year. The ORR was 36.7%, with a CR rate of 3.3%, and a pathologic response rate of 77.8%, with pathologic CR rate of 14.8%.^40^ Histopathology revealed dense tumor‐infiltrating lymphocytes in 95.2% of responders versus 50.0% of nonresponders (*p* < .05).^40^

#### Canerpaturev (HF10, C‐REV)

Canerpaturev, an oncolytic HSV‐1 mutant, combined with ipilimumab in a phase 2 melanoma trial produced a 7% ORR at 24 weeks with a manageable toxicity profile (grade ≥3 AEs, 12%).^41^, ^42^ Preclinical data indicated enhanced lymphocyte accumulation in tumor‐draining lymph nodes, supporting immune‐mediated antitumor activity.^41^

#### Coxsackievirus A21 (CAVATAK, V937, gebasaxturev)

Coxsackievirus A21 (CVA21) is a naturally occurring picornavirus that selectively infects melanoma through ICAM‐1 (intracellular adhesion molecule 1) and decay‐accelerating factor.^43^ In the phase 2 CALM study (ClinicalTrials.gov identifier NCT01227551), intratumoral CVA21 monotherapy achieved a 38.6% ORR without grade 34 toxicities.^44^, ^45^, ^46^, ^47^ Combination strategies have produced mixed results.^44^, ^45^, ^46^, ^47^ In the CAPRA phase 1b study (ClinicalTrials.gov identifierNCT02565992), CVA21 plus pembrolizumab produced a 47% ORR with acceptable toxicity (grade ≥3 treatment‐related AEs, 18%).^44^ Whereas, in the neoadjuvant KEYMAKER‐U02 trial (ClinicalTrials.gov identifier NCT04303169), the addition of CVA21 failed to improve the pathologic CR rate (28% vs. 40%) and increased grade 3–4 toxicity (28% vs. 7%).^45^

#### ONCOS‐102

ONCOS‐102 is a GM‐CSF–expressing oncolytic adenovirus.^48^ In a pilot study of 21 patients with anti–PD‐1–resistant, advanced melanoma, ONCOS‐102 plus pembrolizumab achieved a 35% ORR, with 53% showing regression of at least one noninjected lesion (ClinicalTrials.gov identifier NCT03003676).^49^

#### Poliovirus: rhinovirus chimera (lerapolturev)

PVSRIPO is a live, non‐neurovirulent, oncolytic rhinovirus:poliovirus chimera that infects and lyses tumors cells and inflames the TME. In phase 1 trial of treatment‐refractory melanoma, the ORR was 33% among 12 patients (ClinicalTrials.gov identifier NCT03712358).^50^ Tumor analysis indicated that higher intratumoral CD8‐ppsitive T cells correlated with longer PFS (median PFS, 2.3 years vs. 1.6 months).^51^ The randomized phase 2 LUMINOS‐102 trial (ClinicalTrials.gov identifier NCT04577807) completed accrual in 2024.^52^

#### MEDI5395

MEDI 5395 is a GM‐CSF–expressing, oncolytic Newcastle disease virus that has been evaluated with durvalumab in a phase 1 study among solid tumors. Among 39 patients (four with melanoma), a 10.3% PR rate and early signals of PFS benefit were observed,^53^ supporting further tumor‐specific studies.

### Toll‐like receptor agonists as immunotherapeutic agents

Toll‐like receptors (TLRs) are pattern‐recognition receptors that detect microbial and damage‐associated molecular patterns, initiating inflammatory cascades that activate antigen‐presenting cells and promote effector T‐cell differentiation. As cancer therapeutics, TLR agonists modulate the TME by enhancing local T‐cell infiltration and antitumor immunity, offering potential synergy with ICIs.^54^

#### Vidutolimod

Vidutolimod is a CpG‐A oligodeoxynucleotide TLR9 agonist packaged in a virus‐like particle (VLP). The VLP targets plasmacytoid dendritic cells and delivers CpG‐A to endosomal TLR9, inducing interferon gamma (IFN‐γ) and proinflammatory cytokines that enhance T‐cell priming and recruitment.^55^ Mechanistic analyses have demonstrated that vidutolimod can induce immune remodeling through macrophage polarization toward a proinflammatory phenotype rather than classic innate immune agonism.^56^ In the Eastern Cooperative Oncology Group–American College of Radiology Imaging Network trial ECOG‐ACRIN 6194 (ClinicalTrials.gov identifier NCT04708418), neoadjuvant pembrolizumab plus vidutolimod achieved a major pathologic response rate of 81.4% (22 of 27 patients) versus 68% (17 of 25 patients) for pembrolizumab alone, with similar rates of TRAEs.^57^ In a phase 2 study of stage III melanoma, vidutolimod plus nivolumab produced a major pathologic response in 17/31 patients. The median RFS was not reached at 26.5 months. The 1‐year RFS rate was 94% among patients who had a major pathologic response.^56^

#### Tilsotolimod

Tilsotolimod is a synthetic TLR9 agonist that can increase IFN‐γ and expanded local T cells. In the phase 3 ILLUMINATE‐301 trial in patients with anti–PD‐1 refractory, unresectable, stage III/IV melanoma (ClinicalTrials.gov identifier NCT03445533), intratumoral injection of tilsotolimod (up to nine doses only) plus ipilimumab did not improve outcomes (ORR, 8.8% vs. 8.6%; median OS, 11.6 vs. 10 months for the combination vs. ipilimumab alone, respectively). These results contrasted with the prior phase 1/2 results reporting a 22.4% ORR with combination therapy. It is speculated the phase 3 results were influenced by several factors: (1) suboptimal dosing, with only 23.1% of patients completing all nine intratumoral injections; (2) incomplete treatment delivery, with 51.2% of participants receiving less than one half of the prespecified injections; and (3) differing baseline characteristics, with higher percentages of patients who had elevated lactate dehydrogenase and stage M1C disease.^58^

#### Nelitolimod

Nelitolimod is a synthetic TLR9 agonist that has shown promise when used in combination with immunotherapy. In the phase 1b/2 SYNERGY‐001/KEYNOTE‐184 study (ClinicalTrials.gov identifier NCT02521870), 87 patients with unresectable stage III/IV disease received intratumoral nelitolimod (2 or 8 mg) with intravenous pembrolizumab. Of the 87 patients, 79% had stage IV disease, and 71% were treatment‐naive. Treatment was well tolerated. The ORR was 71% of the 2‐mg group versus 49% in the 8‐mg group. Responses were observed at distant metastatic sites. PFS was not reached in the 2‐mg group versus 10.4 months in the 8‐mg group. In addition, the phase 1 PERIO‐01 trial (ClinicalTrials.gov identifier NT04935229) evaluated hepatic arterial infusion of nelitolimod (SD‐101) alone or with ICIs in patients with metastatic uveal melanoma, demonstrating tolerability with only one grade ≥3 AE and primarily low‐grade cytokine‐related effects.^59^

#### NKTR‐262

Unlike previous TLR9 agonists, NKTR‐262 is a TLR7/TLR8 agonist activated by single‐stranded, viral RNA, giving it a distinct viral‐like structure. It was evaluated with bempegaldesleukin (BEMPEG), a CD122‐biased IL‐2 agonist, in the phase 1 REVEAL study. Among 36 patients receiving intratumoral NKTR‐262 with intravenous BEMPEG, the regimen was well tolerated. In metastatic melanoma, disease control was achieved in 41.2% of patients, including two PRs in heavily pretreated individuals.^60^

### Stimulator of interferon genes agonists

Stimulator of interferon genes (STING) agonists represent a promising class of immunotherapeutics that activate innate immunity through cytosolic DNA sensing. The pathway is initiated when cyclic guanosine monophosphate‐adenosine monophosphate (GMP–AMP) synthase (cGAS) detects aberrant cytosolic DNA and generates cyclic dinucleotides that activate STING. Activated STING complexes with and is phosphorylated by TBK1 (TANK‐binding kinase 1), which activates IFN‐regulatory factor 3, driving transcription of type I interferons to enhance antitumor immunity.^61^, ^62^

In tumors with high mutational burden, cGAS–STING activation can enhance immune recognition; whereas, in chromosomal instability–driven cancers, chronic signaling may promote immune suppression and tumor progression.^63^ Clinical efforts to therapeutically target this pathway have been challenged by limited efficacy, systemic toxicity, and paradoxical protumor effects observed with STING agonists. These findings highlight the context‐dependent nature of cGAS–STING signaling and the need for a more nuanced understanding of its role in cancer biology.^64^

#### MIW815 (ADU‐S100)

MIW815, a synthetic STING agonist, demonstrated enhanced antitumor activity with ICIs in preclinical models. In a phase 1b trial with spartalizumab (*n* = 106; 38 with melanoma), the ORR was 13%, with greater activity in ICI‐naive patients, and 7.8% in previously treated melanoma.^65^ Treatment was generally tolerable, although most patients discontinued because of disease progression.^65^

#### MK‐1454

MK‐1454, a cyclic dinucleotide STING agonist, has demonstrated synergistic activity with PD‐1 blockade in preclinical models.^66^ In a phase 1 study of advanced solid tumors and lymphomas, intratumoral MK‐1454 showed no activity as monotherapy, whereas combination therapy with pembrolizumab achieved an ORR of 16% in cutaneous disease and 4% in visceral disease, with manageable toxicity and dose‐limiting events in approximately 10% of patients.^67^ Subsequent phase 2 studies in other tumor types produced modest ORRs (range, 4%–5%).^68^

#### MK‐2118

MK‐2118, a noncyclic dinucleotide STING agonist, produced minimal antitumor activity alone and modest activity (range, 4%–6%) when combined with pembrolizumab in a phase 1 trial across intratumoral and subcutaneous dosing formulations.^69^

### Cytokine‐based therapies

#### Tavokinogene telseplasmid

Tavokinogene telesplasmid (TAVO) is a plasmid encoding IL‐12 that is delivered intratumorally with electroporation. TAVO increases intratumoral IL‐12, initiates IFN‐γ–driven T‐helper 1 cell (Th1) effector mechanisms, and licenses dendritic cells to prime cytotoxic effectors. It strengthens the C‐X‐C motif chemokine ligand 9 (CXCL9)/CXCR3 axis to create a local chemokine gradient that draws CXCR3‐positive/CD8‐positive T cells and natural killer cells into the TME, supporting a productive immune response and improving sensitivity to PD‐1 blockade.^70^

TAVO‐EP (IL‐12 electroporation) has demonstrated variable but promising activity across clinical settings. In the phase 2 KEYNOTE‐695 trial of anti–PD‐1–refractory melanoma (ClinicalTrials.gov identifier NCT03132675), TAVO‐EP plus pembrolizumab did not meet its prespecified ORR end point (range, 10.2%–18.8%) but showed durable benefit in responders, with a median response duration of 25.5 months and a median OS of 22.7 months.^11^

In contrast, a phase 2 neoadjuvant study (ClinicalTrials.gov identifier NCT04526730) combining TAVO‐EP with nivolumab reported encouraging activity, with a 70% ORR, an 88.9% major pathologic response rate, and a 66.7% pathologic CR rate, highlighting its potential to induce robust systemic and intratumoral immune responses in earlier stage disease.

#### Daromun

Daromun combines two antibody cytokine fusions, L19IL2 and L19TNF. The L19 antibody binds to an alternatively spliced extra‐domain B of fibronectin, which is produced only in cells undergoing neoangiogenesis (i.e., tumor cells). This allows for direct targeting of IL‐2 and tumor necrosis factor to the tumor bed and synergistic activation of a robust immune response that can lead to increased immune cell infiltration and activity.

Daromun has demonstrated promising activity in multiple trials. In the phase 3 PIVOTAL study of resectable stage III melanoma (ClinicalTrials.gov identifier, NCT02938299), neoadjuvant intralesional daromun achieved a 21% pathologic CR rate and significantly improved median RFS (16.7 vs. 6.9 months; HR, 0.59) and distant metastasis–free survival (HR, 0.60) compared with surgery alone. Treatment was well tolerated, with grade ≥3 TRAEs in 14% of patients, and additional studies are ongoing.^71^

#### Aldesleukin

Aldesleukin (synthetic high‐dose IL‐2) was approved in 1998 for metastatic melanoma. Early intravenous trials produced an ORR of 16% but substantial toxicity, including capillary leak, sepsis, and 2% treatment‐related deaths.^72^ To limit systemic effects, intratumoral IL‐2 has been examined. In a phase 2 study of 24 patients, the CR rate in injected sites was 63%, with an ORR of 91% across 245 injected lesions, and toxicities were mostly low‐grade AEs.^73^ A second study in 48 patients reported a CR rate of 69% in all treated metastases, often lasting greater than 6 months, with only grade 1–2 toxicities. Responses were largely confined to injected lesions, with minimal activity in noninjected disease.^74^

### Messenger RNA–based intratumoral treatments

#### Messenger RNA‐2752

Messenger RNA (mRNA)‐2752 is a lipid‐nanoparticle formulation encoding human OX40L, IL‐23, and IL‐36γ that is designed to inflame the TME by enhancing co‐stimulation and proinflammatory cytokine signaling, thereby improving antigen presentation and T‐cell infiltration.^75^, ^76^ Early phase studies reported on target induction of encoded proteins in injected lesions, downstream cytokine changes, manageable safety alone or with durvalumab, and preliminary antitumor activity in refractory tumors, including melanoma.^75^, ^77^, ^78^ Ongoing evaluation in one trial (Clinicaltrials.gov identifier NCT03739931) is defining dosing, schedules, and expansion cohorts across solid tumors.^4^, ^74^

#### mRNA‐4157

mRNA‐4157/V940 is a personalized lipid‐nanoparticle formulation encoding up to 34 patient‐specific neoantigens, administered with pembrolizumab in the adjuvant setting after complete resection of high‐risk, cutaneous melanoma.^79^ In KEYNOTE‐942, a randomized phase 2b trial (ClinicalTrials.gov identifier NCT03897881), patients with stage IIIB–IV disease were assigned 2:1 to receive either mRNA‐4157 plus pembrolizumab or pembrolizumab alone in 3‐week cycles. The combination improved RFS at the primary analysis and, with a median follow‐up of approximately 35 months, a sustained benefit, with a 2.5‐year RFS rate of 74.8% versus 55.6%.^79^, ^80^ Combination therapy reduced the risk of recurrence or death by 49% and improved distant metastasis‐free survival by 62%.^79^, ^80^ Safety was manageable.

### Double‐stranded RNA/RIG‐I–like receptor agonists

#### BO‐112

BO‐112 is a synthetic double‐stranded RNA mimic that activates TLR3 and MDA5, inducing type I interferon signaling and enhancing antitumor immunity.^10^ In the phase 2 SPOTLIGHT‐203 trial of anti–PD‐1–refractory melanoma (ClinicalTrials.gov identifier NCT04570332), intratumoral BO‐112 plus pembrolizumab achieved an ORR of 25% (10% CRs, 15% PRs) and a disease control rate of 65%.^10^ The median response duration was not reached at 15.5 months, with 85.7% of responses ongoing at 9–12 months. The median PFS was 3.7 months, and the 12‐month and 24‐month OS rates were 63.6% and 53.7%, respectively. The regimen was generally well tolerated.^10^

### Other small molecules

#### Rose bengal (PV‐10)

Rose bengal disodium, PV‐10, is a 10% xanthene dye delivered intralesionally for chemoablation of melanoma. It induces rapid tumor necrosis, releases damage‐associated molecular patterns, including HMGB1 (high‐mobility group box 1 protein), promotes dendritic cell maturation, and can elicit regression in some distal, uninjected lesions.^81^, ^82^, ^83^, ^84^ In a multicenter phase 2 study of patients with stage III–IV melanoma, injected lesions achieved a 51% ORR (26% CRs), noninjected lesions had a 33% ORR, and AEs were mainly grade 1–2 and local.^83^, ^85^ Early combination with pembrolizumab has produced a 67% ORR in PD‐1–naive cohorts and a 29% ORR in ICI‐refractory patients.^83^, ^86^ Validation trials are warranted given the promising preliminary efficacy. Notably, a phase 1b/2 randomized trial has recently concluded.^87^

## DISCUSSION

**FIGURE 1:**
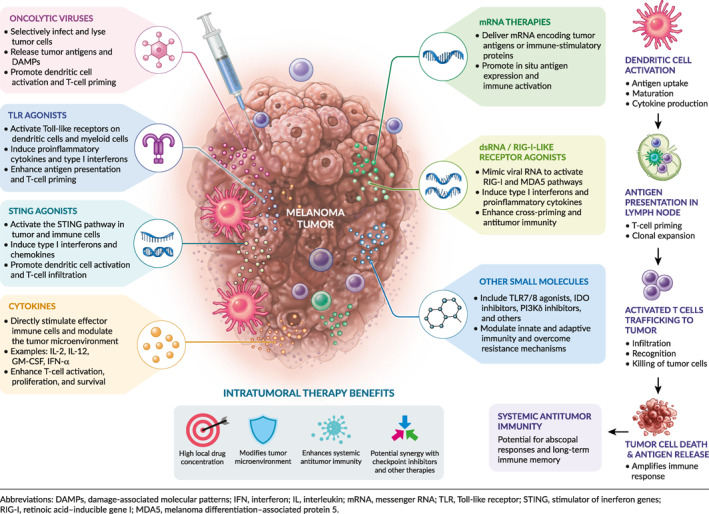
Mechanistic overview and therapeutic rationale for intratumoral therapies in melanoma. Intratumoral therapies deliver immune‐activating agents directly into melanoma lesions to achieve high local drug concentrations, remodel the tumor microenvironment, and promote local and systemic antitumor immunity. Major therapeutic classes include oncolytic viruses, Toll‐like receptor agonists, STING agonists, cytokines, mRNA‐based therapies, double‐stranded RNA/RIG‐I–like receptor agonists, and other small molecules. These approaches can induce tumor cell death and antigen release, activate dendritic cells, enhance antigen presentation in draining lymph nodes, promote T‐cell priming and clonal expansion, and facilitate trafficking of activated T cells back to tumor sites. Through these mechanisms, intratumoral therapies may amplify immune‐mediated tumor killing, generate abscopal responses in noninjected lesions, support long‐term immune memory, and provide rational synergy with immune checkpoint inhibitors and other systemic therapies. RIG, retinoic acid‐inducible gene; STING, stimulator of interferon genes.

Intratumoral therapies have progressed from experimental concepts to clinically validated strategies in melanoma, offering a bridge between locoregional tumor control and systemic immune activation (Figure 1). Agents like T‐VEC and RP1 exemplify the dual mechanism of direct oncolysis and immune priming, establishing proof of concept for this therapeutic class. Newer platforms, including RNA mimetics, cytokine plasmids, and viral or pattern‐recognition receptor agonists, continue to expand the therapeutic landscape, demonstrating activity even in ICI‐refractory disease. Table 1 summarizes key study results as of 2025. These therapies are generally well tolerated, with AEs typically limited to mild, flu‐like symptoms or local inflammation.

**TABLE 1:** Summary of key intratumoral trial results for melanoma.

Treatment type	Trial: Reference	ClinicalTrials.gov identifier	No.	Phase	Agent/s (mechanism of action)	Combination	Patient population	ORR, %	DRR, %	DCR, %	CR, %	Grade ≥3 TRAEs, %
Oncolytic viruses	OPTiM: Andtbacka 2015, 2019^7^, ^8^	NCT00769704	436	3	T‐VEC (HSV‐1 expressing GM‐CSF)	None	Unresectable stage IIIB–IVM1c melanoma	31.5	19.3	76.3	16.9	27.4
	MASTERKEY‐265: Chesney 2023^19^	NCT02263508	692	3	T‐VEC (HSV‐1 expressing GM‐CSF)	Pembrolizumab	Untreated stage IIIB–IV melanoma	48.6	42.2	56.6	17.9	20.7
	Chesney 2023^20^	NCT01740297	198	2	T‐VEC (HSV‐1 expressing GM‐CSF)	Ipilimumab	Untreated stage IIIB–IV melanoma	35.7	33.7	56.1	20.4	45.0
	IGNYTE: Wong 2025^9^	NCT03767348	140	1/2	RP1 (HSV‐1 expressing GM‐CSF and with GLAV‐GP‐R)	Nivolumab	Anti–PD‐1 resistant melanoma	32.9	NR	NR	15.0	12.9
	Cui 2022^39^	NCT01935453	26	1b	OrienX010 (HSV‐1 expressing GM‐CSF)	None	Unresectable stage IIIC–IV melanoma	19.2	NR	53.8	NR	3.8
	Liu 2024^40^	NCT04197882	30	1b	OrienX010 (HSV‐1 expressing GM‐CSF)	Toripalimab	Resectable acral melanoma	NR	NR	NR	14.8	6.6
	Isei 2018^42^	NCT03153085	28	2	Canerpaturev (spontaneous mutant of HSV‐1)	Ipilimumab	Unresectable or metastatic melanoma after anti–PD‐1 therapy	7.0	NR	NR	NR	12.0
	CALM: Andtbacka 2015^46^, ^47^	NCT01227551	57	2	Coxsackievirus A21 (genetically unmodified oncolytic strain)	None	Stage IIIC and IV melanoma	28.1	19.3	NR	NR	0.0
	CAPRA: Silk 2022^44^	NCT02565992	36	1b	Coxsackievirus A21 (genetically unmodified oncolytic strain)	None/ICIs	Advanced melanoma	47.0	74.0	NR	22.0	14.0
	KEYMAKER‐U02: Dummer 2025^45^	NCT04303169	66	1/2	Coxsackievirus A21 (genetically unmodified oncolytic strain)	Pembrolizumab	Resectable stage IIIB–IIID melanoma	32.0	NR	NR	28.0	28.0
	Shoushtari 2023^49^	NCT03003676	21	1	ONCOS‐102 (adenovirus expressing GM‐CSF)	Pembrolizumab	Anti–PD‐1 resistant advanced melanoma	35.0	25.0	NR	5.0	38.0
	Beasley 2021^50^	NCT03712358	12	1	PVSRIPO (poliovirus: rhinovirus chimera)	None	Unresectable, treatment‐refractory melanoma	33.0	NR	NR	16.7	0.0
	Davar 2024^53^	NCT03889275	39	1	MEDI5395 (recombinant Newcastle disease virus expressing GM‐CSF)	Durvalumab	Advanced solid tumors	10.3	NR	30.8	0.0	69.2
TLR agonists	ECOG‐ACRIN EA6194: Tarhini 2025^57^	NCT04708418	57	2	Vidutolimod (TLR‐9 agonist)	Pembrolizumab	High‐risk, resectable melanoma	NR	NR	NR	48.0–71.0	25.0–29.0
	Davar 2024^56^	NCT03618641	31	2	Vidutolimod (TLR‐9 agonist)	Nivolumab	High‐risk, resectable melanoma	45.2	NR	NR	45.1	20.6
	ILLUMINATE‐301: Diab 2024^88^	NCT03445533	481	3	Tilsotolimod (TLR‐9 agonist)	Ipilimumab	Anti‐PD‐1 refractory advanced melanoma	8.8	NR	34.5	NR	61.1
	ILLUMINATE‐101: Babiker 2022^89^	NCT03052205	54	1	Tilsotolimod (TLR‐9 agonist)	None	Refractory solid tumors	0.0	NR	34.3	0.0	47.0
	ILLUMINATE‐204: Haymaker 2021^90^	NCT03445533	62	I/II	Tilsotolimod (TLR‐9 agonist)	Ipilimumab	Anti‐PD‐1 refractory melanoma	22.4	NR	71.4	4.1	48.4
	SYNERGY‐001/KEYNOTE‐184: Ribas 2025^91^	NCT02521870	86	1b/2	Neltilimod (synthetic CpG‐ODN agonist of TLR9)	Pembrolizumab	Anti–PD‐1–naive, advanced melanoma	48.8–75.6	NR	NR	9.8–20.0	60.2
	REVEAL: Diab 2020^60^	NCT03435640	36	1	NKTR‐262 (TLR 7/8 agonist)	Bempegaldesleukin	Advanced solid tumors	NR	NR	41.2	NR	NR
STING agonists	Meric‐Bernstam 2023^65^	NCT03172936	106	1b	MIW815 or ADU‐S100 (STING agonist)	Spartalizumab	Advanced solid tumors	10.4	NR	29.2	0.9	13.2
	Harrington 2018^92^	NCT0301076	60	I	MK‐1454 (STING agonist)	Pembrolizumab	Advanced/Metastatic Solid Tumors or Lymphomas including melanoma	NR	NR	20.0–48.0	0.0	9.0–14.0
	Luke 2025^69^	NCT03249792	140	I	MK‐2118 (noncyclic dinucleotide STING agonist)	Pembrolizumab	Advanced solid tumors	3.8–6.8	NR	NR	1.6‐1.9	11.0–23.0
Cytokines	KEYNOTE‐695: Algazi 2020,^11^ Killmurray 2023, ^93^ Fernandez‐Penas 2021, ^94^ Kähler 2025 ^95^	NCT03132675	98	II	TAVO (IL‐12 plasmid with electroporation)	Pembrolizumab	Stage III/IV melanoma	10.2	8.2	NR	4.1	4.8
	Hasanov 2021^96^	NCT01856023	29	IV	Aldesleukin (synthetic IL‐2)	Ipilimumab	Patients with metastatic melanoma	50.0	NR	83.0	17.0	31.0
mRNA	KEYNOTE‐942: Khattak 2023,^97^Olson 2022^98^	NCT03897881	157	II	mRNA‐4157 (mRNA encoding up to 34 patient‐specific tumor neoantigens)	Pembrolizumab	Resected, high‐risk stage IIIB/IIIC/IIID and IV cutaneous melanoma	NR	NR	NR	NR	NR
dsRNA/RIG‐I‐like receptor agonist	SPOTLIGHT‐203:Márquez‐Rodas 2020, 2025^10^, ^99^	NCT04570332	40	II	BO‐112 (synthetic dsRNA nanoplexed with polyethylenemine)	Pembrolizumab	Anti–PD‐1–resistant melanoma	25.0	NR	67.5	10.0	38.1
Other small molecules	Thompson 2014^85^	NCT00521053	80	2	PV‐10 (synthetic red dye)	None	Refractory, metastatic melanoma	51.0	NR	69.0	26.0	15.0

Abbreviations: anti–PD‐1, anti–programmed cell death 1; CpG‐ODN, CpG oligodeoxynucleotide; CR, complete response; DCR, disease control rate; DRR, durable response rate; dsRNA, double‐stranded RNA; ECOG‐ACRIN, Eastern Cooperative Oncology Group‐American College of Radiology Imaging Network; GLAV‐GP‐R, a fusogenic glycoprotein on vusolimogene oderparepvec allowing for increased cell to cell cytotoxicity; GM‐CSF, granulocyte‐macrophage colony–stimulating factor; HSV‐1, herpes simplex virus type 1; ICIs, immune checkpoint inhibitors; IL‐5, interleukin 5; mRNA, messenger RNA; NCT, ClinicalTrials.gov identifier; NR, not reported; OrienX010, genetically modified herpes simplex virus type 1–encoding human granulocyte‐macrophage colony–stimulating factor; ORR, objective response rate; PV‐10, rose bengal disodium; RIG‐1, retinoic acid‐inducible gene 1; RP1, vusolimogene oderparepvec; STING, stimulator of interferon genes; TAVO, tavokinogene telseplasmid; TILs, tumor‐infiltrating lymphocytes; TLR, Toll‐like receptor; TRAEs, treatment‐related adverse events; T‐VEC, talimogene iaherparepvec.

The incidence of lower rates of significant systemic immune toxicity makes intratumoral therapy promising in immunologically challenging patients. Clinical activity without significant morbidity has been observed in select cohorts, including solid organ transplantation recipients^100^ and patients with underlying autoimmune conditions.^101^, ^102^ Intratumoral therapy can offer a less invasive alternative to surgery in early stage melanoma or more advanced, unresectable patients in whom surgery would be challenging.^103^ Although several intratumoral platforms have shown early promise, current evidence is largely derived from small, early phase studies, and efficacy should be interpreted cautiously pending results from randomized trials.

Despite their promise, intratumoral therapies face practical and technical challenges that may influence trial outcomes. Feasibility is limited by lesion accessibility, procedural complexity (particularly for visceral injections), and variability in injection technique. The absence of standardized, image‐guided protocols and operator‐dependent differences introduce heterogeneity that can confound efficacy and safety assessments. These factors complicate cross‐trial comparisons and regulatory interpretation, highlighting the need for standardized procedural guidelines, operator training, and improved patient selection and biomarker‐driven trial design.

Several limitations temper the efficacy of oncolytic therapy. Negative mechanistic data underscore the pre‐existing and treatment‐induced antiviral neutralizing antibodies that can rapidly restrict viral spread and persistence, whereas immune editing and heterogeneous intratumoral viral replication can limit uniform immune priming.^104^ In addition, although innate immune agonists are often incorporated to enhance antitumor immunity, these agents may also skew macrophage polarization toward immunosuppressive phenotypes within the TME, thereby counteracting therapeutic benefit.^105^ Collectively, these resistance mechanisms highlight persistent biologic barriers that must be addressed to achieve durable clinical responses.

Comparative effectiveness considerations are important when situating intratumoral oncolytic therapies within the treatment landscape, particularly for melanoma refractory to anti–PD‐1–based and BRAF/MEK inhibitor–based regimens. In the heavily pretreated setting, anti–PD‐1/anti–LAG‐3 therapy (nivolumab plus relatlimab) has demonstrated modest clinical activity, with ORRs of approximately 9%–12%.^106^ TIL therapy can produce durable responses in selected patients but is limited by logistical complexity, the need for specialized centers, treatment‐related toxicity, and patient‐specific factors, such as tumor accessibility or disease progression during manufacturing.^107^ In the absence of head‐to‐head comparative studies, available evidence suggests that intratumoral approaches may offer localized disease control with a generally favorable tolerability profile. Currently, these strategies are best considered complementary to established systemic therapies, underscoring the need for prospective studies to better define their optimal role in clinical practice.

Future progress will depend on refining delivery techniques, successfully integrating intratumoral agents with checkpoint blockade, examining combination approaches (including strategies with targeted therapies), treating patients in the appropriate disease setting, adopting more accurate response markers in trials to assess treatment efficacy (i.e., intratumoral RECIST),^108^ and identifying predictive biomarkers to tailor treatment. Although cytokine‐based and mRNA‐based approaches have demonstrated deep pathologic responses in the neoadjuvant setting, translation into the advanced, metastatic setting remains uncertain. Informative preclinical models will be key to prioritize strategies for testing in patients. Beyond therapeutic benefit, intratumoral therapy also serves as a translational platform to study immune activation and resistance in vivo. With continued clinical innovation and standardization, intratumoral immunotherapy is poised to become an increasingly integral component of multimodal melanoma care.

## AUTHOR CONTRIBUTIONS

**Vincent T. Ma**: Conceptualization, data curation, formal analysis, funding acquisition, investigation, methodology, project administration, resources, supervision, validation, writing–original draft, and writing–review and editing. **Alyssa K. Steimle**: Conceptualization, data curation, formal analysis, investigation, methodology, resources, validation, writing–original draft, and writing–review and editing. **Janmesh D. Patel**: Data curation, formal analysis, investigation, methodology, resources, software, writing–original draft, and writing–review and editing. **Caroline Burkey**: Data curation, investigation, resources, validation, writing–original draft, and writing–review and editing. **Justin C. Moser**: Formal analysis, methodology, validation, and writing–review and editing. **Asad Javed**: Investigation, writing–original draft, and writing–review and editing. **Hibba tul Rehman**: Formal analysis, writing–original draft, and writing–review and editing. **Mustafa Ege Seker**: Investigation and writing–review and editing. **Alexander Birbrair**: Writing–review and editing. **Orhan S. Ozkan**: Investigation and writing–review and editing. **Mark R. Albertini**: Writing–original draft and writing–review and editing. **Yana G. Najjar**: Writing–original draft and writing–review and editing. **Rajan P. Kulkarni**: Conceptualization, writing–original draft, and writing–review and editing.

## CONFLICT OF INTEREST STATEMENT

Vincent T. Ma reports institutional research funding from AIQ Solutions, Astellas Pharma, C4 Therapeutics, Fujifilm, Immuneering, Regeneron, Immunocore, Innate Pharma, Jounce Therapeutics, Krystal Biotech, Marengo Therapeutics, Merck, Natera, Pfizer, Replimune, Seagen, Top Alliance BioScience, and Y‐mAbs Therapeutics; personal/consulting or advisory fees from Bristol Myers Squibb Company, Delcath, Incyte, Ideaya Biosciences Inc., Immunocore, Intellisphere, LLC, Partner Therapeutics, Inc., Regeneron Pharmaceuticals, Replimune, Targeted Oncology and Y‐mAbs Therapeutics; and gifts from the Conquer Cancer Foundation and the Society for Immunotherapy of Cancer outside the submitted work. Justin C. Moser reports institutional grants/contracts from Adaptimmune, Agenus, Alpine Immune Sciences, Amgen, BioEclipse Therapeutics, BrightPeak Therapeutics, Fate Therapeutics, FujiFilm, Genentech, IDEAYA Biosciences, Immatics, ImmuneSensor Therapeutic, IOnctura, Iovance Biotherapeutics, Istari Oncology, Nektar Therapeutics, NovoCure, Orionis, Repertoire Immune Medicines, Replimune, Rubius Therapeutics, Senhwa Biosciences, Simcha Therapeutics, Sparx Therapeutics, Storm Therapeutics, Strand Therapeutics, Synthorx Inc., Trishula Therapeutics, T‐Scan Therapeutics, the University of Arizona, Werewolf Therapeutics, and Y‐mAbs Therapeutics; other grants/contracts from iOnctura, NovelWise Pharma, and Trutino Biosciences; institutional consulting fees from Werewolf Therapeutics; personal/consulting fees from Adagene, Amunix, Boxer Capital, Bristol Myers Squibb Foundation, Genome Insight, Imaging Endpoints, Immunocore, Incyte, Iovance Biotherapeutics, IQVIA, Novotech, Oberland Capital, Pfizer, Red Arrow Therapeutics, Replimune, Sun Pharma, Thirona Bio, and Vilya; payment or honoraria for lectures, presentations, speakers’ bureaus, writing, or educational events from Caris Life Sciences, Castle Biosciences, Curio Science, Daiichi Sankyo/Lilly, Horizon CME, IDEOlogy Health, Immunocore, and TGen; and patent US PCT/US24/27766 (granted) outside the submitted work. Orhan S. Ozkan reports personal/consulting fees from Delcath, General Electric, and Neuvascular; and owns stock in Elucent Medical outside the submitted work. Mark R. Albertini reports institutional grants/contracts from Anyxis Immuno‐Oncology GmbH, Aperion Biologics, Array BioPharma, Bristol Myers Squibb Company, Erasca Inc., Merck Sharp & Dohme, and Nektar outside the submitted work. Yana G. Najjar reports institutional grants/contracts from Bristol Myers Squibb Company, Merck, Pfizer, and Replimune; personal/consulting fees or honoraria from Bristol Myers Squibb Company, Immunocore, InverVenn Bio, Merck, Novartis, Pfizer, Replimune, Roche, Sun Pharma, and Therakos; and support for attending meetings and/or travel from Isatari outside the submitted work. Rajan P. Kulkarni reports support for professional activities from Replimune outside the submitted work. The remaining authors disclosed no conflicts of interest.
